# Usefulness of Macroscopic On-Site Evaluation Using a Stereomicroscope during EUS-FNB for Diagnosing Solid Pancreatic Lesions

**DOI:** 10.1155/2022/2737578

**Published:** 2022-01-18

**Authors:** Takuya Ishikawa, Eizaburo Ohno, Yasuyuki Mizutani, Tadashi Iida, Kota Uetsuki, Jun Yashika, Kenta Yamada, Noriaki Gibo, Toshinori Aoki, Kunio Kataoka, Hiroshi Mori, Yoshihisa Takada, Hidekazu Takahashi, Hironori Aoi, Katsuyuki Kato, Takeshi Yamamura, Naomi Kakushima, Kazuhiro Furukawa, Masanao Nakamura, Yoshiki Hirooka, Hiroki Kawashima

**Affiliations:** ^1^Department of Gastroenterology and Hepatology, Nagoya University Graduate School of Medicine, Nagoya, Aichi, Japan; ^2^Department of Endoscopy, Nagoya University Hospital, Nagoya, Aichi, Japan; ^3^Department of Pathology and Clinical Laboratories, Nagoya University Graduate School of Medicine, Nagoya, Aichi, Japan; ^4^Department of Gastroenterology and Gastroenterological Oncology, Fujita Health University, Toyoake, Aichi, Japan

## Abstract

**Methods:**

We reviewed a total of 60 consecutive patients who underwent both S-MOSE and rapid on-site cytopathological evaluation (ROSE) during EUS-FNB between July 2019 and October 2020, and the usefulness of S-MOSE in comparison with histology was evaluated. A 22-gauge Franseen needle was used to perform EUS-FNB in all patients, and only the specimens obtained by the first pass were evaluated. The final diagnosis was based on the surgical specimen or the clinical course consistent with the EUS-FNB results.

**Results:**

The final diagnoses of the 60 patients included 45 patients with pancreatic ductal adenocarcinoma, 6 with autoimmune pancreatitis, 4 with mass-forming pancreatitis, 1 with pancreatic metastasis, 2 with pancreatic neuroendocrine tumor, and 2 with intraductal papillary mucinous carcinoma. The histological diagnostic accuracy of the first pass of EUS-FNB was 83.3% (50/60). The agreement between the S-MOSE and the histological diagnosis was 90% (54/60). The positive predictive value of S-MOSE for histological diagnosis was 90.7%, which can be an indicator of when to stop the EUS-FNB procedure. There were no immediate or delayed adverse events reported after the FNB based on the chart and medical visit history review.

**Conclusion:**

In the EUS-FNB of SPLs, S-MOSE can be an alternative to ROSE for specimen evaluation and has the potential to shorten the procedure time.

## 1. Introduction

Endoscopic ultrasound-guided fine-needle aspiration (EUS-FNA) is a widely used technique for pancreatic tissue sampling [1]. In recent years, with the development of new treatments, such as immune checkpoint inhibitors and gene panel tests, tissue sampling has become increasingly important in pancreatic cancer. Several new core needles have been developed to obtain adequate amounts of tissue, and the technique using these new needles is called endoscopic ultrasound-guided fine-needle biopsy (EUS-FNB). Rapid on-site cytopathological evaluation (ROSE) is a useful method of specimen evaluation for EUS-FNA [2], and the use of ROSE is believed to reduce the number of punctures and improve diagnostic performance. However, the number of facilities that can perform ROSE is limited, and it does not necessarily lead to a reduction in procedure time. As an alternative to ROSE, macroscopic on-site evaluation (MOSE) has been reported to provide a similar diagnostic yield to conventional EUS-FNA in the absence of ROSE but with fewer passes [3]. However, there is no established method for MOSE. In the present study, we aimed to investigate the usefulness of MOSE using a stereomicroscope (S-MOSE) during EUS-FNB with a 22-gauge Franseen needle for the diagnosis of solid pancreatic lesions (SPLs).

## 2. Methods

### 2.1. Study Design

This was a single-center, retrospective study performed at Nagoya University Hospital. It was performed with the approval of the Ethics Committee of Nagoya University Hospital, the content of the research was described, and the contact information for nonparticipation was provided in an opt-out format on the website of our hospital (approval number: 2019-0310). The study was performed in accordance with the ethical standards stated in the 1964 Declaration of Helsinki and its later amendments or comparable ethical standards associated with Grants-in-Aid for Scientific Research (grant no. JP20K12689) support.

### 2.2. Patients

We reviewed a total of 60 consecutive patients in whom EUS-FNB was performed using a 22-gauge Franseen needle (Acquire, Boston Scientific Co., Natick, MA, USA) for SPLs, and the specimens obtained were evaluated by both S-MOSE and ROSE between October 2019 and October 2020 at our institute. Although multiple needle passes were performed on some patients, only the specimen obtained from the first pass was evaluated to reduce selection bias.

### 2.3. EUS-FNB Procedure

The EUS procedure was performed by 2 experts with more than 10 years (EO and TIs) of experience or by trainees under their supervision using a linear-array endoscope (EG 580UT, Fujifilm Co., Ltd., Tokyo, Japan, or GF-UCT260, Olympus Co., Ltd., Tokyo, Japan). While the patient was under conscious sedation, the EUS scope was inserted orally. The lesion was first carefully observed in B-mode and then in color Doppler mode before puncture to confirm that no major vessels were in the needle pathway. A 22-gauge Franseen needle (Acquire) was used to perform EUS-FNB in all patients. The Franseen needle is a newly developed core needle with three novel symmetrical heels. After the needle was inserted into the lesion, the stylet was slowly withdrawn (dry slow-pull technique) as the sample was obtained for all needle passes. In principle, the number of passes was determined based on the findings of ROSE until tumor cells were confirmed, with a maximum of 3 passes. All patients who underwent EUS-FNB were routinely admitted to the hospital for at least 24 hours after the procedure, and short-term adverse events (AEs) were assessed during admission. Additional follow-up information was obtained by review of medical charts and contact with the referring physicians up to 30 days after EUS. Any AEs were recorded and compared according to the lexicon for endoscopic AEs advocated by the American Society of Gastrointestinal Endoscopy [4].

### 2.4. Specimen Processing for EUS-FNB

**Figure 1:**
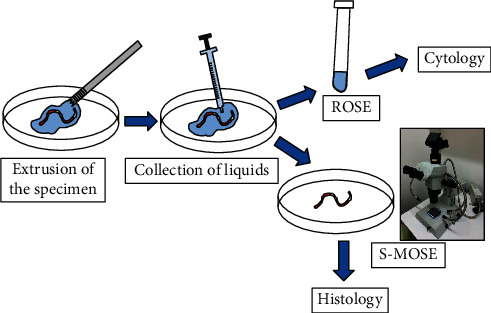
Specimen processing with an endoscopic ultrasound-guided fine-needle biopsy. After each pass, the specimen was extruded from the needle onto a Petri dish with saline. The liquid components around the specimen were aspirated with a syringe and submitted for rapid on-site cytopathological evaluation (ROSE) and cytology. The remaining solid specimens were immediately evaluated under a stereomicroscope and then submitted for histological examination.

The specimen processing workflow for EUS-FNB at our facility is shown in Figure 1. The specimen was extruded from the needle onto a Petri dish using saline. The liquid components around the specimen were then aspirated with a syringe and submitted to ROSE and cytology. The remaining solid specimens were immediately observed under a stereomicroscope, and images were captured. The specimens were then placed in formalin solution for histological examination. All the specimens were processed per needle pass and the hematoxylin-eosin (H&E) staining slides were also made per needle pass.

### 2.5. ROSE Procedure

The submitted liquid component was diluted to 6 ml with saline. Smears were prepared by processing with Auto smear (Sakura Finetek Japan, Co., Ltd., Tokyo, Japan) at 1500 rpm for 5 seconds, promptly spray-fixed (Melcofix®, Merck KGaA, Darmstadt, Germany), and stained with the ultrafast Papanicolaou (UFP) method. Microscopic evaluation was performed inside the endoscopy suite by an experienced cytologist (KKato), and the presence or absence of cell components was immediately reported to the endosonographer.

### 2.6. MOSE Using a Stereomicroscope (S-MOSE)

**Figure 2:**
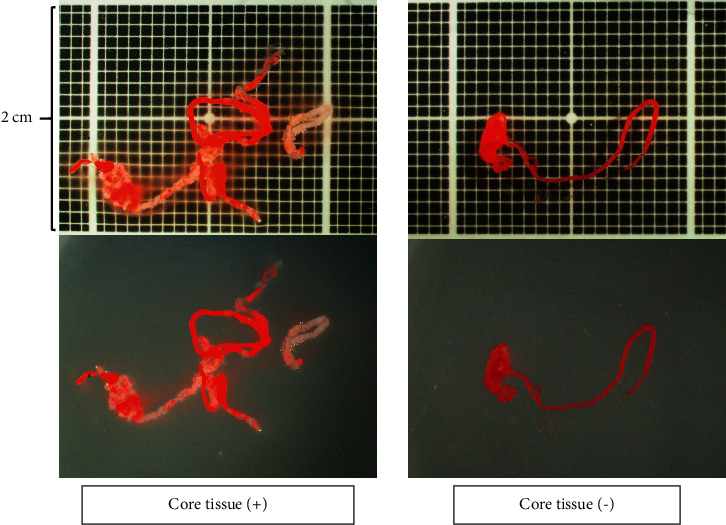
Macroscopic on-site evaluation using a stereomicroscope. The observation screen was set up with a vertical width of 2 cm with a scale of 1 mm behind it. The quality of the specimen is evaluated with nothing behind it.

A high-end zoom stereomicroscope, SZX12 (Olympus Co., Ltd., Tokyo, Japan), was used for S-MOSE. The magnification range was from 7x to 90x (zoom ratio 12.86) with an aperture mechanism that allows a deeper depth of field. To evaluate the specimens under the same conditions as possible, we set up the observation screen so that the vertical width was 2 cm with a scale of 1 mm behind it (Figure 2). The specimen was then observed with nothing behind it, and images were captured to evaluate the quality of the specimen. The evaluation was performed on-site by a single endosonographer (TIs). A specimen was defined as S-MOSE-positive if it contains a portion that was recognizable as white/tan core tissue, while reddish blood clots may also be present. A specimen was defined as S-MOSE-negative if it contains scant or no core tissue or only reddish blood clots. To assess the reliability of the S-MOSE evaluation, the stereomicroscopic images were independently reviewed after EUS-FNB procedures by another reader (EO) who was blinded to the patient history and clinical, radiologic, and histologic information.

### 2.7. Histology Evaluation

**Figure 3:**
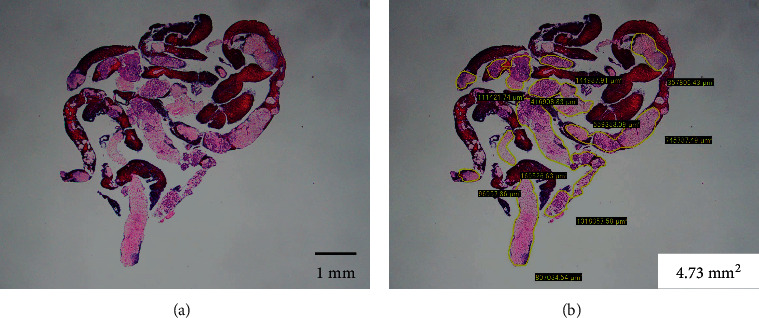
Evaluation of the tissue area using imaging software. (a) Hematoxylin and eosin staining of a gross specimen obtained with endoscopic ultrasound-guided fine-needle biopsy, viewed in a low-power field. (b) Measuring the area of the tissue specimen, excluding the blood clots, using imaging software (CellSens).

After formalin fixation, the specimens were embedded in paraffin, sectioned, and subjected to H&E staining and appropriate immunostaining according to the suspected diagnosis. All histological diagnoses were performed by two pathologists (SY and EI) who specialize in the pancreatobiliary field at Nagoya University Hospital. The final diagnosis was based on the surgical specimen or the clinical course consistent with the results of EUS-FNB with a minimum 6-month follow-up. Histological diagnosis of EUS-FNB specimens was classified into one of the following 5 categories: malignant, suspicious, atypical, benign, and inadequate. Malignant lesions, such as pancreatic cancer, were considered positive if they were malignant or suspicious for malignancy. For benign lesions, such as mass-forming pancreatitis, in addition to showing atypical or benign on histology, a positive diagnosis was made if it was confirmed that the lesion did not worsen during the 6-month follow-up period. With regard to autoimmune pancreatitis (AIP), the final diagnosis was made based on the International Consensus Diagnostic Criteria (ICDC) [5]. The total area of the specimen obtained with each needle was also measured and compared under a photomicroscope using imaging software (CellSens, Olympus Co., Ltd., Tokyo, Japan) based on our previous reports (Figure 3) [6, 7].

### 2.8. Evaluation Items

The evaluation items were as follows: (1) histological diagnostic accuracy of EUS-FNB, (2) Agreement between S-MOSE and histological diagnosis, (3) evaluation of tissue sample area and diagnostic accuracy, (4) comparison between S-MOSE and tissue sample area, and (5) AEs.

### 2.9. Statistical Analysis

All statistical analyses were performed using SPSS Statistics 25.0 (SPSS, Inc., Chicago, IL, USA) software. The Mann-Whitney *U* test was used to compare tissue samples and diagnostic accuracy. Continuous parameters are presented as the median (interquartile range, IQR). The cutoff value of the amount of specimen required for positive histological diagnosis was assessed by receiver operating characteristic (ROC) curve analysis, and the area under the ROC curve (AUC) was calculated. A *P* value of less than 0.05 was considered statistically significant. Interobserver variability of S-MOSE findings was assessed by calculating the kappa coefficient after the two readers had made their individual assessments. Agreement was defined as minor (kappa coefficient, 0.01–0.20), fair (0.21–0.40), moderate (0.41–0.60), good (0.61–0.80), or excellent (0.81–1.00), beyond chance.

## 3. Results

### 3.1. Histological Diagnostic Accuracy of EUS-FNB

The median age of 60 patients was 67 years (IQR 60–72.75), and males accounted for 68.3%. The median size of the target lesions was 25.5 mm (IQR 20–37), with a similar proportion of puncture sites from head to tail (Table 1). The final diagnoses for 60 patients were pancreatic ductal adenocarcinoma in 45 patients, AIP in 6 patients, mass-forming pancreatitis in 4 patients, pancreatic metastasis in 1 patient, pancreatic neuroendocrine tumor in 2 patients, and intraductal papillary mucinous carcinoma in 2 patients. The median number of needle passes was 2 (IQR 1–2). The histological diagnostic accuracy of EUS-FNB by the first pass was 83.3% (50/60) (Table 2).

### 3.2. Agreement between S-MOSE and Histological Diagnosis

According to the evaluation by S-MOSE, 90% of the specimens (54/60) had white/tan core tissue and were judged as S-MOSE-positive, and 10% (6/60) had scant/no core tissue and were judged as S-MOSE-negative. S-MOSE evaluation could correctly predict the histological diagnosis positivity in 54 of 60 cases, namely, the agreement between S-MOSE and histological diagnosis was 90% (Table 3). Kappa coefficients showed that interobserver agreement evaluated by two readers was excellent for S-MOSE evaluation (kappa coefficient = 0.832).

### 3.3. Evaluation of the Tissue Sample Area and Diagnostic Accuracy

**Figure 4:**
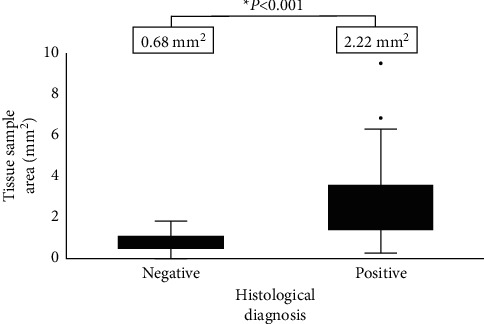
Evaluation of the tissue sample area and diagnostic accuracy. The tissue area was significantly larger in patients who were correctly diagnosed by histology (2.22 mm^2^ vs. 0.68 mm^2^, *P* < 0.001).

The median tissue area of all the FNB specimens was 1.8 mm^2^ (IQR 0.991–2.91), and the tissue area was significantly larger in patients who were correctly diagnosed by histology (2.22 mm^2^ vs. 0.68 mm^2^, *P* < 0.001) (Figure 4). In the ROC curve analysis, the AUC was 0.9, and the cut-off value of the tissue area for positive histological diagnosis calculated based on Youden's index was 1.3 mm^2^, with a sensitivity of 79.2% and a specificity of 91.7% (since the lumen width of a 22-gauge needle is approximately 0.4 mm, a tissue area of 1.3 mm^2^ can be translated to a tissue length of 3.25 mm). Of the 6 cases diagnosed as AIP (all type 1), we were able to obtain histological findings of level 2 or higher (2 cases of level 1 and 2 cases of level 2) based on ICDC in 4 patients. The median tissue area collected from these 4 patients was 2.35 mm^2^ (range 2.03–2.84), while the remaining 2 had tissue volumes of 0.2 mm^2^ and 0.66 mm^2^, indicating that a core tissue size of at least 2 mm^2^ (5 mm in length) is desired to obtain a histological diagnosis of AIP.

### 3.4. Comparison between S-MOSE and Tissue Sample Area

The median tissue area was significantly larger in patients who were S-MOSE-positive (1.95 mm^2^ vs. 0.49 mm^2^, *P* < 0.001). In the ROC curve analysis, the AUC was 0.898, and the cut-off value of the tissue area for S-MOSE-positive calculated based on Youden's index was 0.98 mm^2^, with a sensitivity of 83.3% and a specificity of 83.3%

### 3.5. Adverse Events (AEs)

There were no immediate or delayed (within 30 days) AEs reported after FNB based on chart and medical visit history review.

## 4. Discussion

In this study, we proposed a novel evaluation method for MOSE using a stereomicroscope, and S-MOSE could correctly predict the histological diagnosis positivity in 90% of the cases. EUS-FNA for the pancreas was first reported by Vilmann et al. in 1992 [1], and there have been many reports showing its usefulness and safety. To date, many efforts have been made to improve the diagnostic performance of EUS-FNA [2, 8–10]. Iglesias-Garcia et al. [2] reported that ROSE significantly improved the sensitivity (96.2 vs. 78.2%, *P*=0.002) and accuracy (96.8 vs. 86.2%, *P*=0.013) of cancer diagnoses and was associated with a significantly lower number of inadequate samples (1.0 vs. 12.6%, *P*=0.002) and a lower number of needle passes (3.5 ± 1.0 vs. 2.0 ± 0.7, *P* < 0.001). However, the ROSE requires the presence of a cytopathologist and additional cost and time for slide staining and interpretation. One of the other innovations to improve the diagnostic performance is the shape of the needle tip, such as needles with a reverse bevel design (Echo Tip ProCore, Cook Medical, Bloomington, USA) [11], six cutting edge surfaces (SharkCore, Medtronic Corp. Boston, MA, USA) [12], and three symmetrical heels called a Franseen needle (Acquire) [6, 13], which was used in the present study. Due to the advent of these core needles, much more tissue can be obtained with a smaller number of needle passes, and some reports suggest that using ROSE to reduce the number of needle passes may be needless in the era of EUS-FNB [11, 13–15].

MOSE is a recently introduced alternative to ROSE and shows high accuracy for use in histological diagnosis. Iwashita et al. [16] first assessed the efficacy of MOSE in estimating the adequacy of histologic core specimens obtained by EUS-FNA using a standard 19-gauge needle for solid lesions and concluded that a macroscopic visible core of ≥4 mm on MOSE could be an indicator of specimen adequacy and could improve diagnostic yield.

Since then, there have been several reports showing the usefulness of MOSE in EUS-FNB using core needles [17–20]. However, no established method for MOSE has been reported to date. Kaneko et al. [17] assessed 77 consecutive patients who underwent EUS-FNB using 22-gauge Franseen needles for pancreatic masses and measured the visible cores using a ruler during MOSE. In cases where the visible cores were fragmented, the fragments were gathered and aligned using a 23-G injection needle. In their study, the diagnostic accuracy of EUS-FNB per pass was 92%, and visible core lengths >10 mm independently affected the correct diagnosis. In their report, a ruler was used to measure the length of the tissue, which seems to be a useful method, but the process of collecting the fragmented tissue while excluding blood clots could be somewhat complicated and time-consuming. Oh et al. [19] reported the efficacy of using filter paper to increase the adequacy of histologic core specimens while minimizing blood contamination. Seventy-nine consecutive patients with an intraabdominal mass underwent EUS-FNB, and the diagnostic accuracy, sensitivity, and specificity were 94.5%, 94.3%, and 100%, respectively. The use of filter paper could be a useful option; however, since filter paper is white, we assumed that it may be difficult to recognize the whitish tissue in some samples by macroscopic observation.

**Figure 5:**
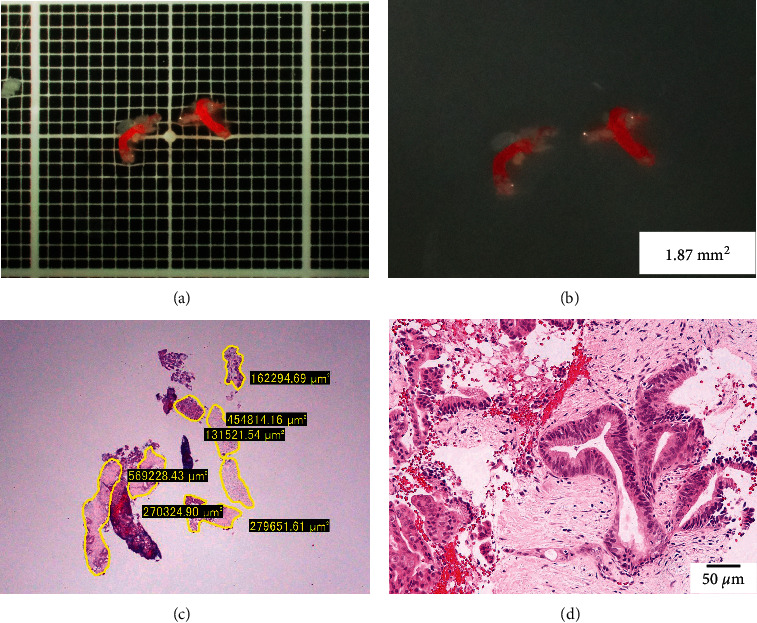
An example where stereomicroscopic observation was considered useful in determining the presence of core tissue. (a, b) A specimen obtained from a mass in the pancreatic body, observed under a stereomicroscope with (a) and without (b) a black scale. The specimen is relatively small, which is difficult to macroscopically evaluate in detail, but under a stereomicroscope, white core tissue is observed in addition to red blood clots. (c) Measurement using imaging software (CellSens) shows that 1.87 mm^2^ of tissue was collected. (d) Photomicrograph showing a component of atypical cells with enlarged nuclei in the fibrous stroma, consistent with ductal carcinoma of the pancreas.

Okuwaki et al. [21] used a stereomicroscope to estimate the cutoff length for the visible white core required for the pathological diagnosis of subepithelial lesions from samples obtained using a 22-gauge Franseen needle, and the diagnostic results were significantly better with cutoff lengths ≥4 mm, similar to the results of the present study. We believe that the use of a stereomicroscope in MOSE has 3 advantages. First, compared with normal macroscopic observation, simply magnifying the image makes it easier to recognize the blood clots and core tissue (Figure 5). Second, the stereomicroscope is placed immediately next to the EUS room, and observation can be performed immediately after specimen collection, which may shorten the examination time. Finally, all the specimens can be evaluated under the same conditions by aligning the magnification using a scale under stereomicroscope observation. This increases objectivity, facilitates measurement of tissue length, and leads to image-based analysis in the future.

As with previous reports, there was a positive correlation between tissue volume and histological diagnostic performance. Based on the ROC curve analysis, the cutoff value of the amount of tissue required for correct histological diagnosis was 1.3 mm^2^, which can be translated into 3.25 mm. Therefore, if a core tissue of 3.25 mm or more can be identified by S-MOSE, it can be assumed that a histological diagnosis is likely to be obtained in most cases. In addition, there was a positive correlation between S-MOSE positivity and tissue volume with the cutoff value of 0.98 mm^2^, supporting the high agreement between S-MOSE and histological diagnosis. However, we included a variety of histological types in the present study and the difficulty of histological diagnosis is different among the diseases. AIP is one of the most difficult presentations, and it is expected that more tissue will be needed for diagnosis. In fact, in all 4 cases in which AIP was diagnosed using EUS-FNB specimens in the present study, core tissue at least 2 mm^2^ in size were collected. Thus, the amount of tissue needed may vary depending on the disease. Moreover, in the treatment of pancreatic cancer, the development of new treatments, such as immune checkpoint inhibitors and gene panel tests, has increased the amount of tissue required. It is important to be able to predict the amount of tissue at the time of EUS-FNB, and S-MOSE can be a useful method in this respect.

In the present study, S-MOSE showed higher sensitivity (98% vs. 68%) but lower specificity (60% vs. 50%) than ROSE (Table 3). Since a liquid sample was used for ROSE and a core sample was used for MOSE, it could be difficult to make a simple comparison; however, these differences may be due to the pathological features of pancreatic cancer, which is frequently accompanied by fibrous tissue, desmoplastic changes, and pancreatitis caused by pancreatic cancer itself. [22, 23] This would probably be recognized as a visible core on MOSE, which may lead to lower specificity. However, the PPV for both ROSE and S-MOSE neared 90% (89.5% and 90.7%, respectively), indicating that both methods had similarly high clinical utility, given the main purpose of the two methods to determine when to stop the procedure.

This study has several limitations. First, the number of patients included in the study was small. Further prospective studies with a larger number of patients are necessary to confirm these results. Second, evaluation by S-MOSE is subjective and lacks objectivity. In recent years, artificial intelligence (AI) has made remarkable progress, and its usefulness in the fields of gastroenterology and endoscopy is attracting attention [24, 25]. We hope that AI will help in MOSE diagnosis in the near future. Finally, the final diagnoses were determined according to not only the surgical specimen but also the EUS-FNB results, which might cause misdiagnosis, even with a minimum follow-up period of 6 months.

In conclusion, MOSE using a stereomicroscope is an alternative specimen evaluation method to ROSE and can be an indicator of when to stop the EUS-FNB procedure; thus, the S-MOSE has the potential to shorten the procedure time.

## Figures and Tables

**Table 1:** Patient characteristics.

	*n* = 60
Age, median (IQR)	67 (60–72.75)
Sex, male, *N* (%)	41 (68.3)
Size of the lesion, median (IQR) (mm)	25.5 (20–37)
Targeted area in the pancreas, *n* (%)	
Head	21 (35)
Body	18 (30)
Tail	13 (21.7)
Uncinate process	8 (13.3)
Number of passes, median (IQR)	2 (1-2)
Final diagnosis, *n* (%)	
Pancreatic ductal adenocarcinoma	45 (75)
Autoimmune pancreatitis (type 1)	6 (10)
Mass-forming pancreatitis	4 (6.7)
Pancreatic metastasis	1 (1.7)
Pancreatic neuroendocrine tumor	2 (3.3)
Intraductal papillary mucinous carcinoma	2 (3.3)

IQR: interquartile range.

**Table 2:** Clinical outcomes of EUS-FNB.

Histological findings on EUS-FNB specimens	Final diagnosis	
	Malignant	Benign
Malignant^*∗*^	41	0
Benign^*∗∗*^	9	9
Inadequate	0	1
Total	50	10

Sensitivity	82%	
Specificity	90%	
Accuracy	83.3%	

EUS-FNB: endoscopic ultrasound-guided fine-needle biopsy; ^*∗*^two cases showed findings suspicious for malignancy; ^*∗∗*^two cases showed findings atypical.

**Table 3:** ROSE and S-MOSE in comparison with histology.

		Histology					Histology		
		Positive	Negative	Total			Positive	Negative	Total
ROSE	Positive	34	4	38	S-MOSE	Positive	49	5	54
	Negative	16	6	22		Negative	1	5	6
	Total	50	10	60		Total	50	10	60
	Sensitivity	68%				Sensitivity	98%		
	Specificity	60%				Specificity	50%		
	Accuracy	66.7%				Accuracy	90%		
	PPV	89.5%				PPV	90.7%		
	NPV	27.3%				NPV	83.3%		

ROSE: rapid on-site cytopathological evaluation, S-MOSE: macroscopic on-site evaluation using a stereomicroscope, PPV: positive predictive value, and NPV: negative predictive value.

## Data Availability

The data used to support the findings of this study are available from the corresponding author upon request.
